# Psychiatric Outcomes of Subthalamic Nucleus Deep Brain Stimulation: A Systematic Review of Short- and Long-Term Effects

**DOI:** 10.3390/brainsci15060566

**Published:** 2025-05-25

**Authors:** Mattia Vittorio Pomes, Giordano D’Urso, Ilaria Bove, Luigi Maria Cavallo, Lorenzo Della Ragione, Carmela Palmiero, Francesco Perrotta, Felice Esposito, Teresa Somma

**Affiliations:** 1 Section of Psychiatry, Department of Neuroscience, Reproductive and Dental Sciences, University of Naples Federico II, 80131 Naples, Italy; mavipo93@gmail.com (M.V.P.); lorenzo.dellaragione@libero.it (L.D.R.); francsperrotta@yahoo.it (F.P.); 2 Department of Mental Health and Addiction, Psychiatric Service of Diagnosis and Care-ASL Rome 5, 00019 Tivoli, Italy; 3 Department of Neurosciences and Reproductive, Division of Neurosurgery, Federico II University of Naples, 80131 Naples, Italy; ilariabove90@gmail.com (I.B.); luigimariacavallo@gmail.com (L.M.C.); carme.palmiero@gmail.com (C.P.); esposito.felice@gmail.com (F.E.); teresa.somma85@gmail.com (T.S.)

**Keywords:** deep brain stimulation, subthalamic nucleus, Parkinson’s disease, obsessive-compulsive disorder, psychiatric effects, mood disorders, apathy, impulsivity, electrode location, systematic review

## Abstract

**Background/Objectives**: Deep Brain Stimulation (DBS) of the subthalamic nucleus (STN) is a widely used intervention for Parkinson’s disease (PD) and obsessive-compulsive disorder (OCD). While motor and OCD symptom benefits are established, increasing evidence highlights psychiatric side effects. The underlying mechanisms involve stimulation parameters, electrode positioning, and medication adjustments. This systematic review aims to evaluate the short-term and long-term psychiatric effects of STN-DBS and identify influencing factors. **Methods**: A systematic literature search (PubMed, Scopus, Web of Science, Embase; 2015–2024) was conducted following PRISMA guidelines. Studies examining psychiatric effects of STN-DBS in PD or OCD, reporting quantitative/qualitative psychiatric measures, and specifying stimulation parameters were included. Risk of bias was assessed using the Newcastle-Ottawa Scale (NOS) for observational studies and the Cochrane Risk of Bias Tool for randomized controlled trials (RCTs). **Results**: A total of 16 studies met the inclusion criteria, with sample sizes from 10 to 149 patients and short- to long-term follow-ups (up to 17 years). Short-term effects included transient hypomania, euphoria, increased impulsivity (especially with medial STN stimulation), and sometimes anxiety reduction. Long-term effects showed a tendency towards apathy and depression (apathy increased significantly in one large cohort), particularly linked to ventromedial STN stimulation or dopaminergic medication reduction. Impulse control disorders (ICDs) improved long-term in one study following medication reduction, while impulsivity slightly worsened in another. Verbal fluency decline was commonly reported, though global cognition often remained stable. Psychiatric outcomes (mood/apathy, attention/memory) depended on stimulation location within STN subregions. Higher total electrical energy delivered (TEED) correlated with depressive trait shifts in one study. **Conclusions**: STN-DBS has complex psychiatric consequences. Electrode positioning, stimulation parameters (including location within STN subregions and possibly TEED), and medication adjustments significantly influence outcomes. Careful patient selection, preoperative psychiatric screening, optimized programming targeting specific STN subregions, and cautious medication management are essential to minimize psychiatric risks while maximizing therapeutic benefits for motor and OCD symptoms.

## 1. Introduction

Deep brain stimulation (DBS) of the subthalamic nucleus (STN) has become a standard surgical intervention for the treatment of Parkinson’s disease (PD), effectively improving motor symptoms and reducing the need for dopaminergic medication [1,2]. However, as clinical experience with STN-DBS has expanded, increasing attention has been given to its effects on psychiatric and cognitive functions [3,4]. While many patients report significant motor benefits, others experience mood disturbances, impulse control issues, apathy, or even psychotic symptoms, raising concerns about the broader impact of DBS on emotional regulation and neuropsychiatric health [1,5]. The intricate relationship between motor improvement and psychiatric side effects underscores the complexity of STN-DBS and highlights the need for a systematic analysis of its psychiatric consequences in both the short- and long-term.

The STN is a crucial component of the basal ganglia-thalamocortical circuit, playing a central role not only in motor control but also in cognition and emotional regulation [4,6,7]. Structurally, the STN is functionally divided into three main regions: the dorsolateral (motor), associative (cognitive), and ventromedial (limbic) zones [4,6]. The dorsolateral STN is primarily responsible for the modulation of motor activity, while the ventromedial STN is extensively connected to the prefrontal cortex, limbic structures, and associative cortices, making it a key regulator of impulsivity, mood stability, and cognitive control [6]. When DBS extends beyond the dorsolateral motor territory, it can influence non-motor circuits, leading to psychiatric alterations [6]. This overlap between motor and affective circuits may explain why STN-DBS, despite its efficacy in controlling motor symptoms, has been linked to depression, mania, anxiety, apathy, and other psychiatric manifestations [1,6].

Short-term psychiatric effects of STN-DBS often include transient hypomanic or manic episodes, which are most prominent in the early postoperative period [5]. These effects are hypothesized to arise from excessive dopaminergic activity, either due to DBS-mediated modulation of basal ganglia circuits or suboptimal medication adjustments [5,7,8]. Over time, however, a different pattern of psychiatric alterations may emerge, with apathy and depression becoming more prevalent in long-term follow-ups [1,2]. This shift has been attributed to a combination of DBS-induced neuroplasticity, maladaptive network adaptations, and the reduction of dopaminergic therapy following successful motor symptom control [1]. One particularly concerning phenomenon is Dopamine Withdrawal Syndrome (DAWS), which can manifest as anhedonia, fatigue, and emotional flattening in patients who undergo a rapid reduction in dopaminergic medication post-DBS [1,3].

The influence of stimulation parameters on psychiatric outcomes is another critical factor. While high-frequency stimulation (>130 Hz) is standard, some studies suggest that lower-frequency stimulation (<100 Hz) may minimize psychiatric side effects [7]. Similarly, the total electrical energy delivered (TEED) [9] and the specific location of active contacts on the DBS electrode appear to influence the likelihood of psychiatric complications [4,6]. Stimulation of the ventromedial STN, for instance, has been correlated with an increased risk of mood instability and emotional dysregulation [1,6].

Beyond Parkinson’s disease, STN-DBS is being explored for treatment-resistant depression (TRD), obsessive-compulsive disorder (OCD) [8,10], and other neuropsychiatric conditions. However, the overlap between motor, cognitive, and affective circuits in the STN makes it a challenging target, necessitating precise patient selection and optimized programming strategies to ensure therapeutic benefit while minimizing psychiatric complications [6,10]. The long-term effects of STN-DBS in these non-motor conditions remain an area of active investigation [8].

Given the expanding role of STN-DBS in movement and psychiatric disorders, a systematic review of its psychiatric effects is crucial for guiding clinical decision-making, optimizing stimulation protocols, and refining patient selection criteria. This review aims to consolidate existing knowledge on short-term and long-term psychiatric effects, focusing on the impact of electrode positioning, stimulation parameters, and postoperative medication adjustments on mood, cognition, and impulse control [1,3,6,11]. Understanding these interactions is essential for improving DBS programming strategies and mitigating psychiatric risks in patients undergoing STN-DBS. The main aim of this work is to systematically analyze the psychiatric effects of STN-DBS in patients with PD and OCD, examining the role of stimulation parameters, electrode positioning, and medication adjustments on both short-term and long-term outcomes, and exploring potential underlying mechanisms.

## 2. Materials and Methods

This systematic review follows the Preferred Reporting Items for Systematic Reviews and Meta-Analyses (PRISMA) guidelines [12]. The research project protocol for this systematic review has been registered on PROSPERO, with reference number CRD420251048651. The methodology is designed to comprehensively evaluate the psychiatric effects of subthalamic nucleus deep brain stimulation (STN-DBS) by systematically identifying, selecting, and analyzing relevant studies.

### 2.1. Search Strategy

A comprehensive literature search was conducted across multiple databases, including PubMed, Scopus, Web of Science, and Embase, to identify studies examining the psychiatric effects of STN-DBS in Parkinson’s disease (PD) and obsessive-compulsive disorder (OCD). The search included studies published between 2015 and 2024. The following search terms were used in various combinations: “subthalamic nucleus deep brain stimulation”, “psychiatric effects” OR “neuropsychiatric effects”, “depression” OR “anxiety” OR “impulsivity” OR “apathy” OR “mood disorders”, “long-term outcomes” OR “short-term outcomes”, “dopamine withdrawal syndrome” OR “DBS psychiatric side effects”, “electrode positioning” OR “stimulation parameters” OR “total electrical energy delivered (TEED)”. Additional manual searches were performed in the reference lists of included articles and relevant review papers.

### 2.2. Eligibility Criteria

Studies were selected based on the following inclusion and exclusion criteria: Inclusion Criteria: Original peer-reviewed research articles analyzing psychiatric outcomes following STN-DBS in PD or OCD. Studies reporting quantitative or qualitative measures of psychiatric symptoms, including depression, anxiety, impulsivity, apathy, or psychosis. Longitudinal studies assessing short-term (≤1 year) and long-term (>1 year) psychiatric outcomes post-DBS. Studies that specified stimulation parameters. Studies with full-text availability in English, Italian, or Spanish. Exclusion Criteria: Case reports (unless providing unique mechanistic insight or AE description not found elsewhere), conference abstracts, and non-peer-reviewed sources. Studies focusing exclusively on motor outcomes without psychiatric data. Articles assessing DBS in brain targets other than the STN. Non-English language publications without an available translation.

### 2.3. Study Selection Process

Two independent reviewers screened the titles and abstracts of all retrieved studies for relevance. Full-text articles meeting the inclusion criteria were then assessed for final eligibility. Disagreements between reviewers were resolved through discussion and consensus or, when necessary, by consulting a third independent reviewer.

### 2.4. Data Extraction

The following information was extracted from the included studies using a standardized form: Study characteristics (authors, year, design, sample size), Patient demographics (age, disease duration, psychiatric history), DBS details (target area within STN [e.g., dorsolateral, ventromedial], stimulation parameters [frequency, voltage, pulse width, TEED]), Psychiatric outcomes (short- and long-term effects on depression, anxiety, apathy, impulsivity, psychosis, cognition), Follow-up duration, and Outcome assessment tools.

### 2.5. Risk of Bias Assessment

The methodological quality and risk of bias for included studies were assessed independently by two reviewers. The Newcastle-Ottawa Scale (NOS) was used for observational studies, evaluating selection, comparability, and outcome assessment. The Cochrane Risk of Bias Tool (RoB 2) was applied for any randomized controlled trials (RCTs), assessing domains such as randomization process, deviations from intended interventions, missing outcome data, measurement of the outcome, and selection of the reported result. Discrepancies were resolved by consensus.

### 2.6. Data Synthesis

The extracted data were synthesized qualitatively. A narrative summary focused on identifying common trends in short-term and long-term psychiatric effects, the influence of stimulation parameters and electrode location, the role of medication adjustments, and reported mechanisms. Meta-analysis was not performed due to the heterogeneity in study designs, populations, outcome measures, and follow-up durations across the included studies.

### 2.7. Ethical Considerations

This systematic review relies exclusively on previously published data; therefore, no direct ethical approval or patient consent was required for this study.

## 3. Results

### 3.1. Study Selection

**Figure 1:**
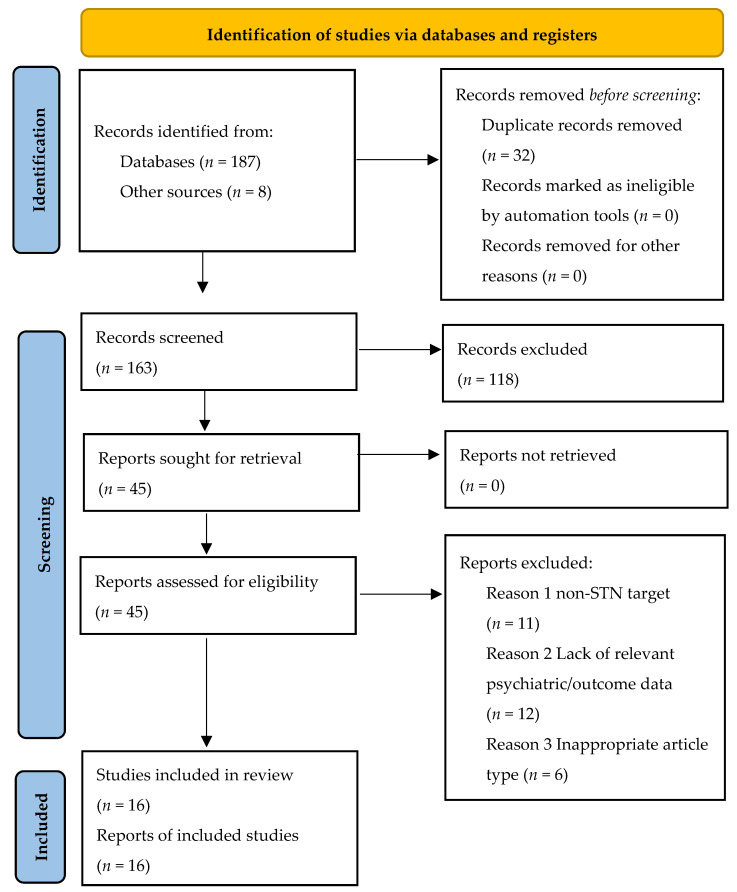
PRISMA 2020 flow diagram illustrating the study selection process. The diagram shows the flow of information through the different phases of the systematic review (Identification, Screening, Eligibility, Included), detailing the number of records identified, included, and excluded at each stage, leading to the final 15 studies included in the qualitative synthesis on psychiatric outcomes of STN-DBS.

The initial database search identified 187 records. An additional 8 records were identified through manual reference list searches, yielding a total of 195 records. After removing 32 duplicates, 163 records were screened based on title and abstract. Of these, 118 were excluded as they did not meet the inclusion criteria (e.g., wrong population, intervention focus on motor outcomes only, different brain target). The full texts of the remaining 45 articles were assessed for eligibility. Twenty-nine full-text articles were subsequently excluded for specific reasons: evaluating the wrong intervention (non-STN target, *n* = 11), lack of relevant psychiatric or specified outcome data (*n* = 12), or inappropriate article type (narrative/non-systematic reviews, *n* = 6). Ultimately, 16 studies [1,2,3,4,5,6,8,9,10,11,13,14,15,16,17,18] fulfilled all inclusion criteria and were included in the qualitative synthesis. The study selection process is detailed in the PRISMA flowchart (Figure 1).

### 3.2. Psychiatric Outcomes

A summary of the included studies is presented in Table 1. Reported psychiatric outcomes varied widely and included depression, anxiety, apathy, mania/hypomania, impulsivity/ICDs, psychosis, cognitive changes (particularly verbal fluency), and changes in quality of life domains related to emotion and social function.

#### 3.2.1. Short-Term Effects (≤1 Year)

Transient psychiatric symptoms during the initial post-operative period and programming phase were common. Hypomania or euphoria, sometimes associated with increased impulsivity or talkativeness, was reported [5,8], particularly linked to stimulation of medial STN contacts [5] or potentially higher voltages [5]. Acute stimulation was also found to modulate impulsivity related to risk; Voon et al. [17] observed that acute STN stimulation decreased risk-taking in a gambling task, despite potentially altering the physiological processing underlying evidence accumulation during decision conflict [17]. Anxiety levels showed variable short-term responses; some studies reported early anxiety reduction (especially physiological anxiety symptoms) [4], while others noted transient anxiety induction during programming [5,8]. Sauerbier et al. [11] found that baseline predictors (ADL, urinary symptoms) could forecast short-term anxiety improvement after STN-DBS.

#### 3.2.2. Long-Term Effects (>1 Year)

Long-term follow-up revealed a complex picture. While initial hypomanic states tended to resolve [5], apathy and depression emerged as significant long-term concerns in several studies [1,2,16]. Abbes et al. [1] found a significant increase in apathy prevalence (from 3% pre-op to 25% post-op) at a mean 6-year follow-up, often independent of depression. Bove et al. [2], in a 17-year follow-up, noted maintained improvement in emotional and social QoL domains, despite worsening parkinsonian symptoms, suggesting complex long-term adaptation, although depression and apathy remained common AEs. Mameli et al. [16] reported increased scores on MMPI-2 scales related to depressive traits one-year post-DBS, despite motor improvement [16]. Conversely, Jiang et al. [9,15] reported stable emotional status (HAMA/HAMD) at 5 and 8 years in their cohorts, and Oner et al. [3] found no significant change in apathy scores (AES) at 6 months post-DBS. Impulse control disorders (ICDs) showed a mixed response. Abbes et al. [1] reported a significant long-term reduction in most ICDs (except eating behavior and hypersexuality) and dopaminergic addiction, likely linked to substantial post-DBS medication reduction. However, transient ICD episodes could still occur during follow-up [1]. Somma et al. [4] observed a slight worsening of impulsivity (BIS-11 total and attentional scores) at 1 year, despite anxiety improvement. The findings by Voon et al. [17] add complexity, suggesting acute STN stimulation might decrease risky choices while potentially affecting other facets of impulsivity related to conflict processing [17]. Cognitive functions were generally stable long-term in most studies [9,15], although a decline in verbal fluency was a frequently reported finding. Ruggiero et al. [18] specifically investigated this, finding a negative correlation between Total Electrical Energy Delivered (TEED) to the left STN and performance on an alternate verbal fluency task, suggesting higher energy delivery might contribute to this decline [18]. For OCD patients, Chabardes et al. [8] reported significant long-term improvement in OCD symptoms (YBOCS) and functioning (GAF), although transient hypomania/anxiety were common AEs. Polosan et al. [10] showed STN-DBS modulated subjective emotional ratings in OCD patients.

### 3.3. Factors Influencing Psychiatric Outcomes

#### 3.3.1. Electrode Location

Evidence strongly suggests that the location of stimulation within the STN influences psychiatric and cognitive outcomes [4,6]. Stimulation of the medial or ventromedial (limbic/associative) STN was associated with acute hypomania/mania [5,8] and potentially worse long-term mood outcomes [1,2], while more dorsolateral (motor) stimulation was generally linked to better psychiatric profiles [4]. Petry-Schmelzer et al. [6] specifically demonstrated location-dependent effects: better mood/apathy improvement correlated with stimulation near the ventral border/sensorimotor STN, while better attention/memory improvement correlated with associative STN stimulation. Stimulation dorsal to the STN was associated with below-average improvement in mood/apathy and attention/memory [6]. Somma et al. [4] also found that more anterior/medial lead positioning negatively influenced psychiatric outcomes (impulsivity).

#### 3.3.2. Stimulation Parameters

Higher voltages (>3 V) and monopolar settings were associated with transient psychiatric symptoms [5]. The role of Total Electrical Energy Delivered (TEED) appears complex and potentially outcome-specific. Mameli et al. [16] found that higher TEED delivered to the right STN correlated with less worsening on MMPI-2 depressive trait scales [16]. Conversely, Ruggiero et al. [18] found that higher TEED delivered to the left STN correlated with worse performance on an alternate verbal fluency task [18]. Somma et al. [4], however, found no correlation between overall TEED and changes in several standard psychiatric symptom scales (HAM-D, BDI, HAM-A, BAI, AES, BIS-11) at 1 year [4]. Lower frequencies (<100 Hz) have been suggested anecdotally or in smaller studies to minimize certain non-motor effects, but this was not a primary focus of the included studies. Acute stimulation settings can also influence decision-making processes related to impulsivity [17].

#### 3.3.3. Medication Adjustments

Significant post-operative reduction in dopaminergic medication (LEDD) was consistently reported [1,2,3,9,15]. While this reduction likely contributed to the improvement of ICDs [1], rapid or excessive reduction was implicated as a major factor in developing post-operative apathy (potentially DAWS) and depression [1]. However, the direct correlation between the magnitude of LEDD reduction and psychiatric outcomes like depression or apathy was not consistently found across all studies [1,3]. Filip et al. [13] found that pre-existing MADD influenced the functional brain response to DBS, independent of medication effects [13].

### 3.4. Risk of Bias Summary

The overall quality of the included studies varied. Observational studies (the majority) assessed with NOS generally showed adequate patient selection but faced moderate to high risk of comparability bias due to uncontrolled variations in stimulation parameters, specific electrode locations, and medication adjustments across centers and patients. Outcome assessment bias was moderate, with variability in the use and reporting of validated psychiatric scales versus clinical observation or different assessment time points. No RCTs focusing primarily on psychiatric outcomes met the inclusion criteria for this review. High-risk studies did not significantly alter the overall qualitative synthesis.

## 4. Discussion

This systematic review synthesizes evidence from 2015 to 2024 on the psychiatric and cognitive outcomes of Subthalamic Nucleus Deep Brain Stimulation (STN-DBS) in patients with Parkinson’s Disease (PD) and obsessive-compulsive disorder (OCD). A summary of the main trends observed across the 16 included studies [1,2,3,4,5,6,8,9,10,12,13,14,15,16,17,18] is presented in Table 2. This table provides a condensed overview of these effects; please refer to the table’s caption and footnotes for a detailed explanation of the symbols and abbreviations used. The findings from the 16 included studies [1,2,3,4,5,6,8,9,10,11,13,14,15,16,17,18] reveal a complex picture, underscoring the intricate interplay between STN modulation, the control of motor or OCD symptoms, and the resulting neuropsychiatric status. Our analysis confirms a pattern where short-term, often transient, activating effects like hypomania, euphoria, or shifts in impulsivity can occur, particularly during initial programming [5,8]. However, the long-term follow-up points towards a heightened risk of apathy and depression [1,2,16], although findings on apathy remain debated [3]. Crucially, these varied outcomes appear significantly influenced by technical and clinical factors, including the precise electrode placement within STN subregions [4,6], the specific stimulation parameters applied (such as voltage, polarity, and potentially Total Electrical Energy Delivered—TEED) [5,16,18], and the necessary postoperative adjustments to dopaminergic medications [1].

Central to understanding these non-motor effects is the dual nature of STN function. Far from being a simple motor relay, the STN serves as a critical node integrating motor, cognitive, and limbic information, channeled through anatomically distinct, yet functionally interconnected, subdivisions [4,6,7]. It receives direct cortical inputs via the hyperdirect pathway, positioning it to rapidly influence downstream processing in response to cognitive and emotional signals [7]. Our findings strongly support this model of the STN as an integrative hub. Stimulation specifically targeting the dorsolateral motor territory generally yields optimal motor benefits [4,6]. Conversely, when stimulation extends into, or primarily affects, the ventromedial (limbic) or associative STN territories, it can precipitate psychiatric or cognitive alterations [3,4,6,18]. This location-dependent effect was clearly demonstrated by Petry-Schmelzer et al. [6], who correlated improvements in mood/apathy with stimulation near the ventral border or sensorimotor STN, while improvements in attention/memory correlated with associative STN stimulation. Similarly, Somma et al. [4] found that more anterior or medial lead positioning could worsen impulsivity, potentially by interfering with the STN’s established role in response inhibition and decision conflict processing [4,17].

The diverse psychiatric sequelae observed post-STN-DBS can thus be conceptualized as consequences of modulating this central integrative node. The common initial transient psychiatric symptoms (TNM), such as hypomania or euphoria [5], may represent an acute over-activation or disruption within the limbic circuits connected via the STN. Acute stimulation effects also extend to complex decision-making processes; the finding by Voon et al. [17] that acute STN stimulation decreased risk-taking in a gambling task, while simultaneously altering physiological correlates of evidence accumulation during conflict, highlights the nuanced role of the STN in different facets of impulsivity [17].

The emergence of long-term apathy and depression represents a significant concern and likely reflects chronic alterations in the delicate balance of these interconnected circuits. While some studies report a significant increase in apathy prevalence over time [1], potentially linked to dopamine withdrawal (DAWS) or direct stimulation effects within the ventromedial STN [1], others found no significant change in apathy scores at 6 months [3], suggesting a complex and multifactorial etiology possibly involving disease progression itself. The inconsistent correlation found between the magnitude of LEDD reduction and changes in apathy or depression scores further supports this complexity [1,3]. Depression outcomes are similarly intricate; while average scores on symptom scales may stabilize in some cohorts [9,15], Mameli et al. [16] documented a worsening on MMPI-2 scales measuring depressive personality traits at one year, illustrating potential discrepancies between symptom reports and underlying personality structure changes [16]. Identified risk factors, such as medication withdrawal, psychiatric history (including MADD impacting network responsiveness [13]), and psychosocial adjustment issues [1], point towards an interaction between DBS effects, disease factors, and individual vulnerability.

The influence of stimulation parameters, particularly TEED, appears similarly multifaceted and context-dependent. While Mameli et al. [16] found that higher TEED delivered to the right STN correlated with less worsening on depressive trait scales, Ruggiero et al. [18] linked higher TEED in the left STN to worse performance on verbal fluency tasks. Yet, Somma et al. [4] found no correlation between overall TEED and changes on several standard psychiatric symptom scales. This suggests TEED’s impact may be specific to the hemisphere stimulated and the functional domain assessed. Simpler parameters like higher voltage (>3 V) and monopolar settings were more consistently linked to transient psychiatric symptoms, likely due to broader current spread [5].

The effects on Impulse Control Disorders (ICDs) also illustrate the interplay between direct DBS effects and medication changes. Improvement in ICDs often follows the substantial LEDD reduction enabled by DBS [1], supporting a primary role for dopaminergic overstimulation in these behaviors. However, the persistence of transient episodes [1] or even worsening scores on specific impulsivity measures like the BIS-11 [4] suggests that DBS itself might contribute, potentially by disrupting the STN’s function in response inhibition or conflict monitoring, as highlighted by Voon et al.’s findings [17]. Anxiety outcomes also remain variable, though some baseline predictors for short-term improvement have been identified [4,11].

Cognitive functions generally appear stable long-term [9,15], with the notable and frequent exception of a decline in verbal fluency, which might be specifically linked to higher TEED in the left STN [18]. The finding that even higher cognitive functions like metacognition seem unaffected by STN-DBS [19] further supports the idea of domain-specific rather than global cognitive effects. In the specific context of OCD, STN-DBS shows therapeutic benefits on core symptoms [8], but carries risks of transient hypomania or anxiety [8], and has been shown to modulate subjective emotional ratings, consistent with its role in affective circuits [10].

This review benefits from a systematic PRISMA-guided approach, a focus on recent literature (2015–2024), the inclusion of both PD and OCD populations, and the consideration of multiple influencing factors (location, parameters, medication). However, the interpretation of these findings is limited by the predominance of observational studies within the recent literature meeting our criteria, the inherent heterogeneity in patient populations, assessment methods, outcome measures, and follow-up durations across studies. A significant further limitation of this review, and indeed of the current body of literature, is the inconsistent and often incomplete reporting of crucial surgical and technical details across the included primary studies [1,2,3,4,5,6,8,9,10,11,13,14,15,16,17,18]. Important factors such as the specific surgical targeting methodologies (e.g., use of microelectrode recording (MER), image-guidance protocols, number of intraoperative electrode passes), whether procedures were staged or simultaneous bilateral, precise trajectory angles, the specific type of stimulating electrode used (including clear documentation of directional capabilities versus non-directional leads), and the detailed methods for postoperative anatomical verification of electrode placement (e.g., specific reconstruction software, atlas-based assessments, or imaging modalities) were not uniformly available or detailed sufficiently for a systematic synthesis. This lack of standardized, granular reporting on such potentially influential variables made it unfeasible to analyze their collective or individual impact on psychiatric outcomes within the scope of the present systematic review. This heterogeneity in reporting likely contributes to the variability in outcomes observed and underscores a challenge in drawing definitive conclusions about the nuanced impact of these specific technical factors. The absence of RCTs primarily focused on psychiatric outcomes also restricts definitive conclusions. Despite these limitations, the synthesized evidence carries significant implications for clinical practice and ethics. The profound impact of STN-DBS on interconnected motor, cognitive, and limbic circuits necessitates meticulous preoperative screening, including detailed psychiatric history and assessment of risk factors [1,13]. Precise surgical targeting aiming for the dorsolateral STN while avoiding encroachment on ventromedial territories appears crucial for minimizing psychiatric side effects [4,5,6]. Postoperatively, careful and individualized adjustment of stimulation parameters, considering factors like voltage, polarity, and potentially TEED [5,16,17,18], coupled with gradual and cautious dopaminergic medication reduction [1,10], is essential. Continuous long-term psychiatric and cognitive monitoring is warranted to detect and manage emergent symptoms like apathy or depression [1]. The ethical considerations are substantial, given the procedure’s potential to alter fundamental aspects of personality and behavior, including mood, motivation, impulsivity, and cognitive style [1,4,5,16,17,18]. Transparent informed consent that thoroughly discusses these potential risks, including the elevated risk of suicide reported in some cohorts [1,8], is paramount. Vigilant balancing of the significant motor or OCD benefits against these potential neuropsychiatric risks is a cornerstone of responsible clinical practice.

Looking forward, several research directions are crucial for refining STN-DBS therapy. Prospective, longitudinal studies employing standardized, multi-domain neuropsychiatric and cognitive assessments with extended follow-up periods [2] are desperately needed to clarify the true long-term trajectory of non-motor symptoms. Randomized controlled trials specifically designed to compare different stimulation parameters or target locations within the STN regarding their non-motor outcomes are essential. Furthermore, to enable more robust and granular synthesis in future systematic reviews, it is imperative that primary studies adopt standardized and comprehensive reporting of surgical and technical details. This includes, but is not limited to, explicit descriptions of targeting strategies, electrode types (distinguishing directional and non-directional systems), precise stimulation parameters including energy delivery metrics, and the methodologies used for VTA reconstruction and anatomical verification of electrode placement. Further research is required to fully elucidate the complex role of TEED [4,16,18], the mechanisms underlying apathy and DAWS [1,3], and to identify reliable predictors for both positive and negative neuropsychiatric outcomes [11,13,14]. Continued investigation into outcomes for OCD [10] and a deeper understanding of how DBS affects specific facets of impulsivity [17] are also warranted. Ultimately, advancing our knowledge of how STN-DBS modulates the interconnected motor, cognitive, and emotional networks will be key to minimizing non-motor risks and maximizing the overall therapeutic benefits for patients with PD and OCD.

## 5. Conclusions

STN-DBS is effective for motor symptoms in PD [2,9,15] and core symptoms in OCD [8], but its use is associated with complex psychiatric and cognitive consequences [1,5,7]. This systematic review of 16 studies [1,2,3,4,5,6,8,9,10,11,13,14,15,16,17,18] highlights a profile including transient activation states (hypomania, impulsivity modulation) [5,8,17] and significant long-term risks, particularly apathy [1] and shifts in depressive personality traits [16], alongside potential effects on verbal fluency [18]. Electrode positioning within STN functional subregions [4,6], stimulation parameters (voltage, potentially TEED) [5,16,18], and medication management [1] are key determinants of these non-motor outcomes.

Optimizing STN-DBS requires a highly individualized approach, integrating meticulous patient selection, precise targeting, tailored programming, and cautious medication adjustments, supported by ongoing psychiatric and cognitive monitoring [1,3,4,6,16,18]. The capacity of STN-DBS to influence mood, motivation, impulsivity, and cognition mandates careful ethical consideration and transparent patient communication. Further research is crucial to refine techniques, understand mechanisms, and minimize non-motor risks while maximizing the overall therapeutic benefits of STN-DBS.

## Figures and Tables

**Table 1:** Summary of Included Studies (N = 16) Evaluating Psychiatric and Cognitive Outcomes of Subthalamic Nucleus (STN) Deep Brain Stimulation (DBS).

Ref.	Population	N Pts (Analyzed)	STN Target Area	Stimulation Parameters (Mean ± SD or Range) ^1^	Key Psychiatric Outcomes Assessed	Follow-Up	Psychiatric Assessment Tools ^2^	Key Psychiatric Conclusions
[1]	PD	69	STN (Presumably dorsolateral)	V: 2.9 ± 0.6 V; Freq: 131.4 ± 24.9 Hz; PW: NS	ICDs, Neuropsychiatric Fluctuations, Apathy, Depression	Mean 6 yrs (3–10)	Ardouin Scale, MINI, BDI, BAI, Starkstein Apathy Scale	Long-term improvement in ICDs/Fluctuations; Apathy significantly increased (25%). Transient psychiatric episodes occurred.
[5]	PD	49 (15 with TNM)	Medial STN/Ni/Zi (Often Monopolar)	V: range 1.15–4.0 V; Monopolar frequent	Transient Non-Motor Psychiatric Symptoms (TNM)	Acute/Transient	Clinical observation, BDI, HAM-D, YMRS, Q-LES	TNM symptoms (voltage-dependent) linked to medial contacts; associated with persistent subclinical depression and lower QoL.
[2]	PD	51	STN	V: ~3 V; Freq: ~130 Hz; PW: ~63 µs (mean @T2)	QoL (PDQL), AE Description (incl. psychiatric)	17.1 yrs	PDQL, UPDRS	QoL (emotional/social) improved despite PD progression. Depression/Apathy common AEs.
[8]	OCD	19	STN Non-Motor (Antero-Medial)	V: 1.1–3.6 V; Freq: 130 Hz; PW: 60 µs	OCD Severity (YBOCS), Functioning (GAF), AEs	24 months	YBOCS, GAF	Significant YBOCS and GAF improvement. Frequent AEs: transient DBS-induced hypomania/anxiety. Two later suicides reported.
[13]	PD (MADD vs. Non)	81	STN	DBS ON vs. OFF (for rs-fMRI)	MADD, Functional Connectivity (rs-fMRI), MADRS	Post-DBS rs-fMRI assessment	MADRS, rs-fMRI	Pre-DBS MADD linked to less fMRI normalization in specific networks post-DBS, despite similar MADRS scores post-op.
[14]	PD	66	STN	NS	ADL Predictors; Psychiatric AEs (delirium)	5 years	MMSE, HDS, S and E, UPDRS	Identifies ADL predictors. Mentions post-op delirium.
[9]	PD (China)	10 (of 20 init.)	STN	V: 2.77 ± 0.49 V; Freq: 121.5 ± 21 Hz; PW: 71.3 ± 12.8 µs (@8 y)	Motor Function, QoL (PDQ-39), Emotion (HAMA/HAMD)	8 years	UPDRS, PDQ-39, HAMA, HAMD, MMSE, MoCA, PDSS-CV	QoL returned to baseline after 3 y; Emotion stable. Low V/Meds maintained.
[15]	PD (China)	10 (of 17 init.)	STN	V: 2.68 ± 0.43 V; Freq: 138.5 ± 19.3 Hz; PW: 75.0 ± 18.2 µs (@5 y)	Motor Function, QoL (PDQ-39), Emotion (HAMA/HAMD)	5 years	UPDRS, PDQ-39, HAMA, HAMD, MMSE, MoCA, PDSS-CV	Cognition/Emotion stable at 5 y. Low V/Meds maintained.
[3]	PD	26	STN	NS	Apathy (AES), Depression (BDI), Anxiety (BAI)	6 months	AES, BDI, BAI, MoCA, PDQ-39	Apathy (AES) did not change significantly at 6 m post-DBS. Conservative LED reduction recommended.
[6]	PD	91	STN (VTA mapping)	Clinically optimized settings	NMSS (total, mood/apathy, attention/memory, sleep)	6 months	NMSS, NMSQ, SCOPA, PDQ-8, VTA Mapping	Mood/apathy, attention/memory, sleep outcomes depend on stimulation location (VTA) within STN.
[10]	OCD	10 (of 12 init.)	STN associative-limbic	DBS ON vs. OFF	Subjective Emotional Ratings (valence/arousal)	Experimental Sess.	Visual Analogue Scales (VAS)	DBS increases positive valence ratings for low-intensity stimuli.
[11]	PD with Anxiety	50 (of 149 tot)	STN	NS	Anxiety (HADS-A), Depression (HADS-D), Predictors	6 months	HADS, NMSS, SCOPA-ADL	Worse baseline ADL and urinary symptoms predict greater anxiety improvement at 6 m.
[4]	PD	14	STN (Position analyzed)	TEED: 0.029 ± 0.001 (mean); Not correlated	Depression, Anxiety, Apathy, Impulsivity, Suicidality	1 year	HAM-D, BDI, HAM-A, BAI, AES, BIS-11, SSI, RFL-48	Anxiety (HAM-A) improved (−29%); Impulsivity (BIS-11) slightly worsened (+9%). Psychiatric outcome related to lead position.
[16]	PD	20	STN	TEED Calculated and Correlated	Depressive Personality Traits (MMPI-2), TEED Correlation	1 year	MMPI-2, TEED Calculation, MADRS, PDQ-8	Higher right STN TEED correlated with less worsening on MMPI-2 D scale. No change in MADRS.
[17]	PD (*n* = 11), OCD (*n* = 4)	15 (acute stim); 25 (iEEG recordings)	STN (Right)	Acute DBS ON vs. OFF (Settings: 60 µs, 130 Hz, Mean V 2.9 ± 0.7)	Impulsivity (Risk-taking), STN Physiology (LFP)	Acute Experimental Sess.	Gambling Task, iEEG	Acute STN-DBS decreased risk-taking but altered STN physiology/evidence accumulation link during conflict.
[18]	PD	24	STN (Left vs. Right TEED analyzed)	TEED Calculated (Mean Left: 1.99 ± 1.04 J/C; Right: 2.06 ± 0.89 J/C)	Verbal Fluency (Cognition), TEED Correlation	Post-op	Alternate Verbal Fluency Battery (AVFB), TEED Calculation	Higher TEED in left STN correlated with worse alternate verbal fluency performance.

Abbreviations: ADL = Activities of Daily Living; AE = Adverse Event; AES = Apathy Evaluation Scale; BAI = Beck Anxiety Inventory; BDI = Beck Depression Inventory; BIS-11 = Barratt Impulsiveness Scale-11; DBS = Deep Brain Stimulation; GAF = Global Assessment Functioning; HADS = Hospital Anxiety and Depression Scale; HAM-A = Hamilton Anxiety Rating Scale; HAM-D = Hamilton Depression Rating Scale; ICD = Impulse Control Disorder; MADD = Mixed Anxiety-Depressive Disorder; MADRS = Montgomery-Åsberg Depression Rating Scale; MINI = Mini-International Neuropsychiatric Interview; MMSE = Mini-Mental State Examination; MoCA = Montreal Cognitive Assessment; NMSS = Non-Motor Symptom Scale; NMSQ = Non-Motor Symptoms Questionnaire; OCD = Obsessive-Compulsive Disorder; PD = Parkinson’s Disease; PDQ = Parkinson’s Disease Questionnaire; PDQL = Parkinson’s Disease Quality of Life Questionnaire; PW = Pulse Width; QoL = Quality of Life; Q-LES = Quality of Life Enjoyment and Satisfaction Questionnaire; RFL-48 = Reasons for Living Inventory-48; rs-fMRI = resting-state functional Magnetic Resonance Imaging; S and E = Schwab and England Scale; SCOPA = Scales for Outcomes in Parkinson’s disease; SSI = Scale for Suicide Ideation; STN = Subthalamic Nucleus; TEED = Total Electrical Energy Delivered; TNM = Transient Non-Motor; UPDRS = Unified Parkinson’s Disease Rating Scale; V = Volts; VAS = Visual Analogue Scale; VTA = Volume of Tissue Activated; YBOCS = Yale-Brown Obsessive Compulsive Scale; YMRS = Young Mania Rating Scale; Zi = Zona Incerta. Notes: ^1^ Stimulation parameters often varied between patients and over time; values provided are examples or means where readily available in abstracts/tables. ^2^ Primary psychiatric/behavioral assessment tools listed; other tools may have been used.

**Table 2:** Summary of General Trends in Psychiatric and Cognitive Outcomes Following STN-DBS (2015–2024).

Psychiatric/Cognitive Domain	Typical Short-Term Outcome (≤1 yr)	Typical Long-Term Outcome (>1 yr)	Key Influencing Factors and Notes
Mood (Depression)	↔/Minor changes reported	↑ Risk/↑ Traits [1,2,16] vs. ↔ Symptoms [9,15]	LEDD Reduction [1], Psychiatric History (MADD) [13], TEED (Right) may ↓ risk [16], Adjustment Issues [1]
Mood (Mania/Hypomania)	↑ (T) Common AE [5,8]	↓ Generally resolves	Location (Ventromedial STN) [5,6,8], Voltage ↑ [5], Monopolar stim [5]. Transient.
Apathy	↔ Generally stable [3]	↑ Increased risk reported [1,2] (Conflicting: ↔ [3])	Location (Ventromedial STN) [1,6], LEDD Reduction (DAWS) [1], Disease Progression
Anxiety	↔ Variable/↓ Specific scales [4], Predictors [11]	↔ Variable/Generally not significantly changed long-term	Lead Position [4], Baseline ADL/Urinary predict improvement [11]. Transient effects possible [5,8].
Impulsivity/ICDs	↑ Impulsivity (T) [4]/↓ Risk-taking (Acute) [17]	↓ ICDs [1]/↑ Impulsivity (BIS-11) [4]	LEDD Reduction primary factor for ↓ ICDs [1]. Lead Position (Ant/Med) ↑ Impulsivity [4]. Differential facet effects [17].
Global Cognition	↔ Stable	↔ Generally Stable [9,15,19]	Major cognitive decline is uncommon if selection criteria are met.
Verbal Fluency	↔/↓	↓ Common finding [16]	Linked to TEED (Left STN) ↑ [18].
OCD Symptoms (OCD Pop.)	↓ Improvement [8]	↓ Sustained Improvement [8]	Non-motor STN target [8]. Modulation of emotional processing [10].

Symbol Legend: ↑: General trend towards increase or worsening of the symptom/trait severity; ↓: General trend towards decrease or improvement of the symptom/trait severity; ↔: General trend towards stability, marked variability between studies, or conflicting results; (T): Effect often reported as transient or occurring mainly during the initial programming phase; [X]: Reference number from the bibliography supporting the indicated finding. Abbreviations: AE: Adverse Event; ADL: Activities of Daily Living; Ant/Med: Anterior/Medial (lead position); DAWS: Dopamine Agonist Withdrawal Syndrome; ICDs: Impulse Control Disorders; LEDD: Levodopa Equivalent Daily Dose; MADD: Mixed Anxiety-Depressive Disorder; OCD: Obsessive-Compulsive Disorder; Pop.: Population; STN: Subthalamic Nucleus; TEED: Total Electrical Energy Delivered (R = Right, L = Left).

## Data Availability

Data sharing is not applicable to this article as no new data were created or analyzed in this study. All data synthesized and discussed are from previously published research articles which are cited in the text and listed in the References section. The PROSPERO registration number for the systematic review protocol is CRD420251048651.
